# Biological Features Implies Potential Use of Autologous Adipose-Derived Stem/Progenitor Cells in Wound Repair and Regenerations for the Patients with Lipodystrophy

**DOI:** 10.3390/ijms20215505

**Published:** 2019-11-05

**Authors:** Keiji Suzuki, Sadanori Akita, Hiroshi Yoshimoto, Akira Ohtsuru, Akiyoshi Hirano, Shunichi Yamashita

**Affiliations:** 1 Department of Radiation Medical Sciences, Atomic Bomb Disease Institute, Nagasaki University, 1-12-4 Sakamoto, Nagasaki 852-8523, Japan; shun@nagasaki-u.ac.jp; 2 Department of Plastic Surgery, Wound Repair and Regeneration, Fukuoka University, School of Medicine, 7-45-1 Nanakuma, Jonan-ku, Fukuoka 814-0180, Japan; akitas@hf.rim.or.jp; 3 Department of Plastic and Reconstructive Surgery, Nagasaki University, 1-12-4 Sakamoto, Nagasaki 852-8523, Japan; hy671117@nagasaki-u.ac.jp (H.Y.); akiyoshi@nagasaki-u.ac.jp (A.H.); 4 Department of Radiation Health Management, Fukushima Medical University School of Medicine, 1 Hikariga-oka, Fukushima 960-1295, Japan; ohtsuru@fmu.ac.jp; 5 Fukushima Medical University School of Medicine, 1 Hikariga-oka, Fukushima 960-1295, Japan; 6 Center for Advanced Radiation Emergency Medicine at the National Institutes for Quantum and Radiological Science and Technology, 4-9-1 Anagawa, Inage-ku, Chiba 263-8555, Japan

**Keywords:** ADSC, lipodystrophy, HIV, HAART, adipogenesis

## Abstract

A paradigm shift in plastic and reconstructive surgery is brought about the usage of cell-based therapies for wound healing and regeneration. Considering the imitations in the reconstructive surgeries in restoring tissue loss and deficiency, stem cell-based therapy, in particular, has been expected to pave the way for a new solution to the regenerative approaches. Limitations in the reconstructive surgeries in restoring tissue loss and deficiency have paved the way for new regenerative approaches. Among them, adipose-derived stem/progenitor cells (ADSCs)-based therapy could be the most promising clue, since ADSCs have pluripotent differentiation capabilities not only in adipocytes but also in a variety of cell types. Accumulating evidences have indicated that the unfavorable development of adipose-tissue damage, namely, lipodystrophy, is a systemic complication, which is closely related to metabolic abnormality. Considering ADSC-based regenerative medicine should be applied for the treatment of lipodystrophy, it is inevitable to ascertain whether the ADSCs obtained from the patients with lipodystrophy are capable of being used. It will be very promising and realistic if this concept is applied to lipoatrophy; one form of lipodystrophies that deteriorates the patients’ quality of life because of excessive loss of soft tissue in the exposed areas such as face and extremities. Since lipodystrophy is frequently observed in the human immunodeficiency virus (HIV)-infected patients receiving highly active antiretroviral therapy (HAART), the present study aims to examine the biological potentials of ADSCs isolated from the HIV-infected patients with lipodystrophy associated with the HAART treatment. Growth properties, adipogenic differentiation, and mitochondrial reactive oxygen species (ROS) production were examined in ADSCs from HIV-infected and HIV-uninfected patients. Our results clearly demonstrated that ADSCs from both patients showed indistinguishable growth properties and potentials for adipocyte differentiation in vitro. Thus, although the number of cases were limited, ADSCs isolated from the patients with lipodystrophy retain sufficient physiological and biological activity for the reconstitution of adipose-tissue, suggesting that ADSCs from the patients with lipodystrophy could be used for autologous ADSC-based regenerative therapy.

## 1. Introduction

Adult stem cells have been used as the promising source of stem cells, which can be applied for cell-based therapies [1,2,3]. Among the adult stem cells, adipose-derived stem/progenitor cells (ADSCs) are the most promising ones, since they can be easily obtained from liposuction aspirates or subcutaneous adipose tissue fragments and expanded in vitro and there are no ethical concerns like human embryonic stem cells [4,5,6,7,8,9]. Furthermore, accumulating evidences have indicated that ADSCs showed multi-lineage differentiation, including classical mesenchymal lineages as well as non-mesenchymal ectodermal and endodermal lineages [1,10,11,12]. Recently, we have applied ADSCs for autologous transplantation therapy for chronic radiation injury [13]. ADSCs were obtained by less invasive lipoaspiration in combination with automatic and aseptic isolation. As ADSCs could be the effective component of transplanted fat tissue, which has been applied to wound repair and regeneration, ADSCs transplantation is expected to show equivalent efficacy to fat tissue transplantation. Thus, ADSCs could be a critical and promising cell population in amending impaired subcutaneous adipose-tissue including lipodystrophy.

Lipodystrophy is characterized by either complete or partial loss of adipose tissue [14]. There are congenital and acquired lipodystrophy, and one representative acquired form occurs in human immunodeficiency virus (HIV)-infected individuals treated with highly active antiretroviral therapy (HAART), and up to 70% of patients receiving HAART are reported to have HIV-associated lipodystrophy. The HAART has been succeeded in inhibiting virus multiplication, and thus, it significantly improves the survival of HIV-infected patients [15,16,17,18]. However, as HIV-infected patients live longer, it became more evident that HAART induced multiple layers of adverse effects including adipose-tissue damage. Adipose-tissue damage manifests as abnormal distribution of adipose tissue, and clinical features of lipodystrophy include peripheral lipoatrophy and central lipohypertrophy [19,20,21,22,23].

It has been reported that lipodystrophy caused by nucleoside reverse transcriptase inhibitors (NRTIs) is related to its effect in mitochondria, which results in apoptosis induction in adipocytes [24,25,26]. Subsequently, NRTIs were substituted with protease inhibitors (PIs), and although PIs showed less effect on mitochondria than NRTIs, it becomes evident that PIs induce endoplasmic reticulum (ER) stress by accumulating unprocessed proteins in adipocytes, which are the inducer for unfolded protein response (UPR)-dependent apoptosis [27]. Thus, PIs have also brought about lipodystrophy in not a small number of the patients receiving HAART [28,29,30,31]. Previously, fat tissue transplantation was applied to lipodystrophy, particularly to facial lipodystrophy [32,33,34]. It efficiently improved facial disfigurement, which resulted in improvement in patient’s quality of life, however, it needed surgical excision of fat tissue. Therefore, ADSCs-based therapy should be more beneficial to the patients with severe lipodystrophy in comparison with the fat tissue transplantation alone, since it is minimally invasive. However, because lipodystrophy is closely related to dysfunction of differentiated adipocytes, and ADSCs are those supply adipocytes in tissue, irreversible and detrimental effects of HARRT on ADSCs could be a possible cause resulting in lipodystrophy [22,23]. Thus, it is inevitable to ascertain whether the ADSCs obtained from the patients with lipodystrophy are capable of being used, which could be tested in vitro.

ADSCs in HIV-infected patients could be obtained from abdomen, thighs, and shoulders, where lipoatrophy was less severe but apparently induced; but, a study has claimed that adipose tissue may be damaged not only by HAART but also by HIV-infection itself through the possible impairment of mitochondrial function [35]. Thus, in order to achieve successful ADSCs-based therapy, the current study aimed at determining whether ADSCs isolated from HIV-infected patients receiving HAART retain sufficient biological and physiological activities for reconstitution of subcutaneous adipose tissue. Total eight ADSCs were established from the subcutaneous lipoaspirates obtained from the donor sites, such as lower abdomen, thighs, buttocks, and shoulders, from three HIV-infected patients and four uninfected patients [36]. Growth properties, adipogenic differentiation, and mitochondrial ROS production were examined in ADSCs from HIV-infected and HIV-unrelated patients ex vivo. Our results clearly demonstrated that ADSCs from HIV-infected patients showed indistinguishable growth properties and potentials for adipocyte differentiation in vitro. Thus, although a number of cases was limited, ADSCs derived from the patients receiving HAART retain sufficient physiological and biological activity for the reconstitution of adipose-tissue, indicating that ADSCs from the patients with lipodystrophy could have sufficient biological potential so that they could be used for autologous ADSCs-based regenerative therapy.

## 2. Results

### 2.1. Isolation and Growth Properties of ADSCs

We have obtained lipoaspirates from three HIV-infected patients who are hemophilic and infected with HIV virus by unheated blood products, diagnosed as lipodystrophy after HAART (Table 1). Lipoaspirates from HIV-infected patients were obtained from the sites such as abdomen, thighs, and shoulders, where lipoatrophy was less severe. Lipoaspirates were also obtained from four patients, who are not related to HIV-infection (Table 1). Lipoaspirates obtained from one patient were mixed and used for ADSCs isolation.

**Figure 1:**
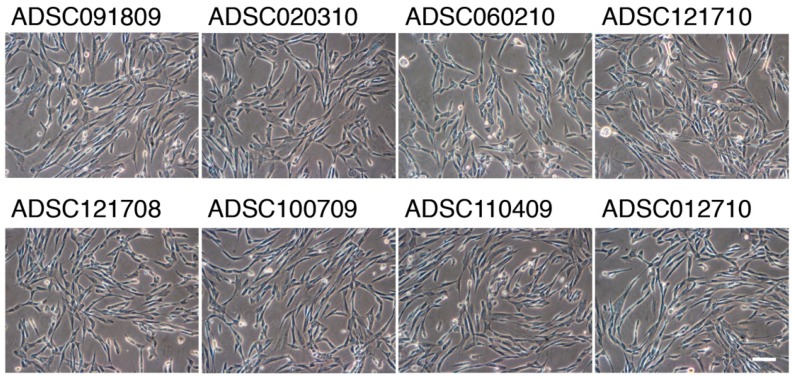
Morphology of adipose-derived stem/progenitor cells (ADSCs) cultures at passage 1 obtained from human immunodeficiency virus (HIV)-infected and HIV-unrelated patients. Exponentially growing ADSCs were cultured in serum free-medium in type I collage-coated flasks. Magnification ×100; the scale bar indicates 100 µm.

Approximately 100 µL of processed lipoaspirates were plated onto type-I collagen-coated flasks and maintained in a serum-free medium. The cells, which clonally expanded were collected and stored as the primary ADSCs. We noticed that the number of colonies formed by the processed lipoaspirates obtained from the HIV-infected patients were about one-tenth of those observed in the lipoaspirates obtained from HIV-uninfected patients. ADSCs showed mesenchymal stem cell-like morphology (Figure 1).

ADSCs isolated from processed lipoaspirates were highly proliferative in serum-free medium, and a plenty of mitotic figures were discerned. We observed no detectable difference in cell morphologies and growth patterns between the ADSCs obtained from the HIV-infected patients and HIV-unrelated patients. We also extensively compared the growth kinetics of ADSCs by cell growth assay, and there was no detectable difference in the growth kinetics of both ADSCs (Table 2).

### 2.2. Adipogenic Differentiation of ADSCs

**Figure 2:**
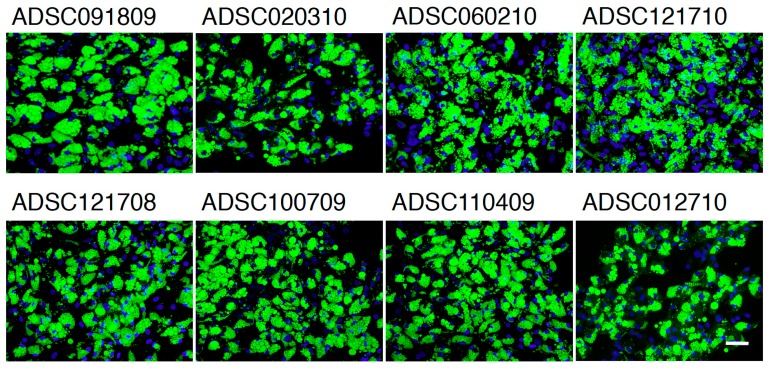
Adipogenic differentiation of ADSCs in culture. ADSCs were stained with 10 µg/mL BODIPY 493/503 and counterstained with 0.1 μg/mL of DAPI. Magnification ×100; the scale bar indicates 100 µm.

Adipogenic differentiation was examined in confluent ADSCs by incubating them in a differentiation medium for 10 days. Multiple tiny lipid droplets became visible by day 5, and they gradually matured during 10 days’ incubation. To quantify adipogenic differentiation, lipid droplets were stained with BODIPY 493/503, a highly sensitive lipophilic fluorescent dye. As shown in Figure 2, multiple lipid droplets were identified in the cytoplasm, whose morphology was similar to that observed in subcutaneous adipocytes. The frequency of differentiation-positive cells were approximately 97% or more in ADSCs from HIV-unrelated patients, and the same differentiation potential was confirmed in ADSCs derived from HIV-infected patients (Table 3). Average fluorescence per cell is compared in Table 3, and we detected significant difference between ADSCs derived from HIV-infected and HIV-unrelated patients.

**Figure 3:**
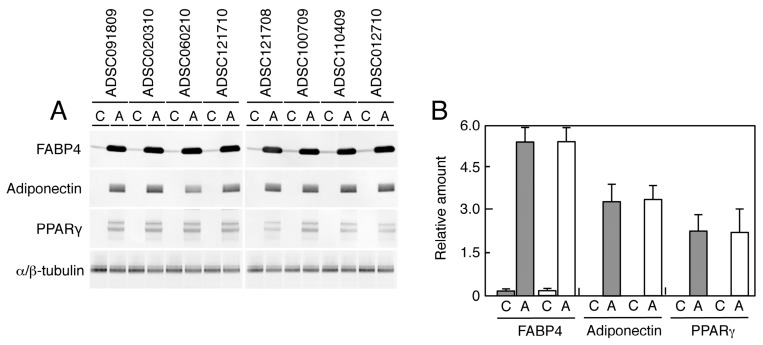
Western blot analysis of adipose-associated proteins. Samples (8 μg for tubulin and 16 μg for other proteins) were subjected to Western blot analysis probed with anti-FABP4, adiponectin, PPARγ, and α/β-tubulin antibodies (**A**). Relative levels of the bands to those of α/β-tubulin were calculated and summarized in (**B**). C—cells grown in the control medium; A—cells differentiated in adipogenic medium. Gray boxes indicate the mean amount observed in the cells from HIV-uninfected patients, while white boxes indicate that observed in the cells obtained from HIV-infected patients. Bars indicate S.D.

Adipogenic differentiation was also confirmed by analyzing the expression of proteins associated with adipocytes. As shown in Figure 3, we identified significant induction of FABP4, adiponectin, and PPARγ in every ADSC differentiated into adipocytes, irrespective of HIV-infection. Average levels of protein expression also show any notable difference.

### 2.3. Mitochondrial ROS Production

**Figure 4:**
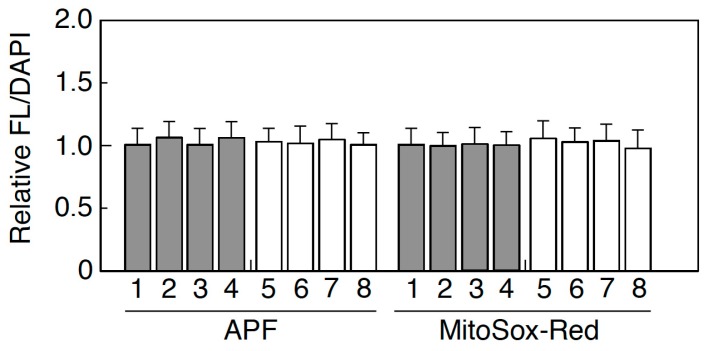
Oxidative stress levels in ADSCs. Intracellular oxidative stress level was measured by APF, and mitochondrial damage was quantified by MitoSox-Red. Gray boxes indicate the mean amount observed in the cells from HIV-uninfected patients, while white boxes indicate that observed in the cells obtained from HIV-infected patients. Bars indicate S.D.

Oxidative stress levels were evaluated by APF and MitoSox-Red in which the former represented intracellular oxidative stress and the latter measured the mitochondrial oxidative stress. As shown in Figure 4, we found no significant increase in ADSCs isolated from both HIV-infected and HIV-uninfected patients. ROS-induced DNA damage was also determined in ADSCs using 53BP1 foci, a surrogate marker for DNA double-strand breaks. While spontaneous DNA damage was detected in all ADSCs, there was no excess amount of DNA damage even in ADSCs obtained from HIV-infected patients.

## 3. Discussion

Several preadipocytes culture systems by which cells are able to be maintained for several passages have been established so far. For example, human preadipocytes were cultured in a medium 199 or DMEM/F-12 medium supplemented with 10% fetal bovine serum (FBS) [10]. ADSCs from lipoaspirates were generally cultured in a DMEM medium containing 10% FBS [8,37]. However, we have noticed that such ADSCs, cultured in a medium containing 10% FBS, rapidly lost their growth potential and exhibited senescence-like morphology. Since fetal bovine serum is well-known to contain some components showing growth inhibitory effects [38], we attempted to find a serum-free culture condition suitable for ADSCs. Our medium was originally prepared for primate embryonic stem cells [39], and it significantly improved the growth of ADSCs in culture, which enabled us to examine their biological and physiological activities.

In HIV-infected patients, subcutaneous adipose-tissue damage is quite obvious among those receiving HAART [19,20,21,22]. In the current study, ADSCs were isolated from the less affected parts of the body of HIV-infected patients. However, the recovery rates of ADSCs from the lipoaspirates obtained from the HIV-infected patients were about one-tenth of those observed in HIV-uninfected patients, indicating that ADSCs obtained from those parts of body were notably damaged. Therefore, it was expected that physiological activity of ADSCs in those parts of the body was compromised. However, our present study clearly demonstrated that there was no detectable difference in the growth kinetics, saturation density, and cloning efficiency between ADSCs obtained from HIV-infected and HIV-uninfected patients (Table 2). We are able to conclude that ADSCs from HIV-infected patients are biologically normal. We then compared the potential of adipogenic differentiation of ADSCs, and we found that the formation of lipid droplets was completely normal in ADSCs obtained from HIV-infected patients, indicating that adipogenic conversion was not compromised in those ADSCs (Figure 2 and Table 3). The conclusion was also confirmed by the protein analysis in Figure 3, in which no difference was detectable in the expression of several proteins involved in adipogenesis. Thus, it is quite obvious that ADSCs from HIV-infected patients retain normal physiological activity. Since previous study has claimed that HIV-infection alone causes lipodystrophy through abrogation of mitochondrial function [32], we have examined whether ADSCs from HIV-infected patients show increased oxidative stress levels or not. As shown in Figure 4, the results clearly showed no detectable change in oxidative stress level in all cases. While mitochondrial damage can occur in vivo, ADSCs isolated from the patients and expanded in culture might regain physiological mitochondrial function, suggesting that ADSCs used for therapy are better to allow time in culture to recover before transplantation.

Recent advances in plastic and reconstructive medicine have allowed autologous stem cell therapy [40,41,42,43,44,45]. As we recently reported [13], it is evident that autologous stem cell therapy is able to retrieve the abrogated function of skin. Furthermore, tissue regeneration using ADSCs involves indirect effects, which accelerate wound healing through secreting growth factors [46,47,48,49,50,51,52]. Thus, autologous ADSC therapy could be a promising solution for subcutaneous adipose-tissue damage, including lipodystrophy in the patients receiving HAART. So far, facial lipodystrophy has been recognized as a common side effect of HAART, and autologous fat grafting is an effective treatment modality. In this study, we proved that ADSCs derived from the patients receiving HAART retain sufficient physiological and biological activity for adipogenic differentiation. In this way, ADSCs from the HIV-infected patients are the ideal sources for autologous stem cell therapy for lipodystrophy. Although we should be cautious as a number of cases examined in this study was limited, ADSCs from the patients with lipodystrophy could have sufficient biological potentials, so that they could be used for autologous ADSCs-based regenerative therapy [53].

## 4. Materials and Methods

### 4.1. Characteristics of Patients

Lipoaspirates were obtained from three HIV-infected patients who are hemophilic and infected with HIV virus by unheated blood products and were diagnosed as lipodystrophy after HAART [36]. The patient #1 and #3, a 30 years-old man, diagnosed HIV positive when he was 15 years old, and the patient #2, a 46 years-of-old man, was notified HIV-positive when he was at the age of 22 years. The patient #4, a 36 years-old man, diagnosed HIV positive when he was 11 years old. Clinically all patients demonstrated the severe lipoatrophy in the naso-labial, melo-labial, temporal and parotid region of their faces. Patient #1 and #3 were severely lean in their torso, while the patient #2 was moderately built in the torso. The patient #4 was well-built in torso but lean in extremities. The body weights of the HIV-infected patients were 56.9 kg, 59.4 kg, and 70.3 kg, respectively and the body mass indexes (BMI) were 19.92, 21.96, and 21.94, respectively. Lipoaspirates from HIV-infected patients were obtained from abdomen, thighs, back, and shoulders where lipoatrophy was less severe. Lipoaspirates were also obtained from four patients, who are not related to HIV-infection. Their mean weight was 48.6 ± 3.93 kg with BMI 22.91 ± 3.303. The mean age of the HIV-infected patients was 35.5 ± 7.55, while it was 72.0 ± 16.1 in HIV-uninfected patients.

### 4.2. ADSCs and Culture

ADSCs were isolated from lipoaspirates as described previously [36]. Briefly, lipoaspirates, obtained from the donor sites, such as lower abdomen, thighs, buttocks, and shoulders, were processed using a Celution^TM^ system (Cytori Therapeutics, Inc, San Diego, CA, USA) based upon the method reported previously [36]. Mixed lipoaspirates isolated from different sites in one patient were used. Approximately 100 µL of processed lipoaspirates were plated onto type I-collagen-coated culture flasks. Since a medium containing 10% FBS promoted cellular senescence of ADSCs, the lipoaspirates were cultured in a serum-free medium originally developed for primate embryonic stem cells (Primate ES medium, ReproCELL, Tokyo, Japan). Clonally expanded cells were collected and stored in liquid nitrogen as the primary ADSCs (passage 0). Exponentially growing ADSCs were maintained by subculturing when they reached to 80% confluence. ADSCs were treated with trypsin/EDTA solution, trypsin-neutralizing solution, and collected by centrifugation for 5 min at 1200 rpm. The cells were resuspended in a fresh medium and 3 × 10^5^ cells were replated onto the T25 flasks (25 cm^2^), while rest of the cells were stored in liquid nitrogen at each passage.

ADSCs were obtained from three HIV-infected patients and four HIV-unrelated patients (Supplementary Table S1). The HIV-infected patient #1 and #3 are the same subject, and received treatments with indinavir, lamivudine (3TC), d4T, and lately, with atazanavir and Euzicom (3TC and abacavir combination) regimens for 15 years. The HIV-infected patient #2 received treatments with AZT, didanosine, 3TC, d4T, and lately, with atazanavir and Tenofovir/emtricitabine for 10 years. The patient #4 received treatments with AZT for 2 years, didaosine for 4 years, lamivudine (3TC) for 12 years, Nelfinavir for 3 years, d4T for 3 years, and Euzicom (3TC and abacavir combination) regimens for 10 years. All HIV-infected patients were diagnosed as HIV-negative by PCR-based assay at the time of isolating ADSCs. The study was approved by the Nagasaki University Hospital ethical committee (Internal Review Board approval No. 08070296, July 22, 2008, Nagasaki University Hospital), and all patients gave written informed consent.

### 4.3. Cell Growth

ADCSs were seeded onto 35 mm culture dishes at a density of 2 × 10^5^ cells/dish. The medium was changed every 2 days, and they were cultured up to 7 days. Cells were collected every day and the numbers of cells were counted by a cell counter (CDA-500, Sysmex, Kobe, Japan). Saturation density was determined as the number of cells at confluence at day 7.

### 4.4. Cloning Efficiency

Exponentially growing ADSCs were collected, counted, and reseeded onto 100-mm culture dishes at a density of 100 cells/dish. The cells were cultured for 10 days without changing a medium before fixation with ethanol. The fix cells were then stained with 3% Giemsa’s solution for 15 min. The numbers of colonies consist of 50 cells or more were counted. Cloning efficiency was determined by dividing the number of colonies by the number of cells plated. Data obtained from three independent experiments were compiled.

### 4.5. Adipocyte Differentiation

Exponentially growing ADSCs were collected, counted the number of cells, and 1 × 10^5^ cells were replated onto 22 mm × 22 mm type I collagen-coated cover glass slips. They were cultured in a serum-free medium until they reached confluence. Then, the culture medium was changed to differentiation medium (DM-2, ZenBio, Inc., Research Triangle Park, NC, USA). They were cultured for another 10 days before fixation with 4% formalin. The fixed cells were stained with 10 µg/mL of BODIPY 493/503 (D-3922, Invitrogen, Carlsbad, CA, USA) for 20 min at room temperature and the nuclei were counterstained with 0.1 mg/mL of DAPI. Accumulation of lipid droplets was determined under a fluorescence microscope (F3000B, Leica, Tokyo, Japan). Digital images were captured and the images were analyzed by FW4000 software (Leica, Tokyo, Japan). Cells containing multiple lipid droplets in more than 50% of the cytoplasm were counted as differentiation positive cells. In order to quantify average fluorescence per cell, the same areas were marked, and the sum of the pixel intensity within the marked area was calculated by FW4000 software, and total green fluorescence was divided by total blue fluorescence obtained by DAPI staining.

### 4.6. Mitochondrial Oxidative Stress

Intracellular oxidative stress level was measured by 3′-(*p*-aminophenyl)-fluorescein (APF). Cells cultured in T25 flasks were washed with PBS and treated with 5 μM APF in PBS for 60 min at 37 °C in a 5% CO_2_ incubator. After the treatment, cells were trypsinized, suspended in PBS at 4 × 10^4^ cells/mL, and green fluorescent intensity was measured by a fluorometer (JASCO, Tokyo, Japan). The excitation and emission wavelengths were set up at 490 nm and 515 nm, respectively.

Mitochondrial damage was quantified by MitoSox-Red. Cells cultured in T25 flasks were washed with PBS and treated with 1 μM MitoSox-Red (Invitrogen) in PBS for 20 min at 37 °C in a 5% CO_2_ incubator. After the treatment, cells were trypsinized, suspended in PBS at 4 × 10^4^ cells/mL, and red fluorescent intensity was measured by a fluorometer (JASCO, Tokyo, Japan). The excitation and emission wavelengths were set up at 400 nm and 580 nm, respectively. The nuclei were counterstained with 0.1 mg/mL of DAPI. Relative fluorescence was calculated by dividing total green or red fluorescence by total blue fluorescence obtained by DAPI staining.

### 4.7. Immunofluorescence

Cells were collected by trypsinization and 5 × 10^4^ cells were replated onto coverslips. The cells were fixed with cold methanol for 10 min on ice followed by washing with 1× PBS^−^. Then, the primary antibodies diluted in TBS-DT (20 mM Tris-HCl, pH 7.6, 137 mM NaCl, 0.1% Tween 20, 125 µg/mL ampicillin, 5% skim milk) were treated for 2 h at 37 °C, followed by the Alexa Fluor-labeled secondary antibodies for 1 h at 37 ˚C. Nuclei were counterstained with 1 μg/mL DAPI. The antibodies used was anti-53BP1 (A300-272A, BioLegend, San Diego, CA, USA), and Alexa Fluor 555-labed anti-rabbit IgG (A21428, Thermo Fisher Scientific, Waltham, MA, USA). Images were captured by fluorescence microscope (DM6000B, Leica, Tokyo, Japan) and analyzed by FW4000 (Leica, Tokyo, Japan).

### 4.8. Western Blotting

Exponentially growing cells were lysed in lysis buffer (50 mM Tris-HCl (pH 7.2), 150 mM NaCl, 1% NP-40, 1% sodium deoxycholate, and 0.1% SDS) containing 1 mM 4-(2-aminoethyl)-benzensulfonyl fluoride hydrochloride. The cell lysate was cleared by centrifugation at 15,000 rpm for 10 min at 4 °C, and then supernatant was used as the total cellular protein. Total protein concentration was determined by the BCA protein assay (Pierce, Rockford, IL). Protein samples (8 or 16 µg) were electrophoresed on SDS-polyacrylamide gel and were electrophoretically transferred to a polyvinyl difluoride membrane in a transfer buffer (100 mM Tris, 192 mM glycine). After overnight incubation with blocking solution (10% skim milk), the membrane was incubated with the primary antibodies, a biotinylated anti-mouse or anti-rabbit IgG antibodies, and streptavidine-alkaline phosphatase. The bands were visualized after addition of nitroblue tetrazolium/5-bromo-4-chloro-3-indolyl phosphate as a substrate. The primary antibodies used in this study are anti-adiponectin (clone 19F1, Abcam Co. Ltd., Tokyo, Japan), anti-FABP4 (Abcam Co. Ltd., Tokyo, Japan), anti-PPARγ (clone 81B8, Cell Signaling technology Japan, Tokyo, Japan), and anti-α/β-tubulin (Cell Signaling technology Japan).

### 4.9. Data Analysis

The data obtained from at least three independent experiments are expressed as mean ± SD. Wilcoxon rank test was used to evaluate the significant difference between the two groups. *P* values of less than 0.05 were considered significant difference.

## Figures and Tables

**Table 1:** Origin of adipose tissue-derived cells.

Cell ID	Patients’ Description	Origin
ADSC091809	HIV-infected patient #1	abdomen, back
ADSC020310	HIV-infected patient #2	thigh, abdomen, shoulder
ADSC060210	HIV-infected patient #3	thigh, abdomen, back
ADSC121710	HIV-infected patient #4	thigh, abdomen
ADSC121708	HIV-uninfected patient	abdomen, buttock
ADSC100709	HIV-uninfected patient	thigh, abdomen
ADSC110409	HIV-uninfected patient	thigh, abdomen
ADSC012710	HIV-uninfected patient	thigh, abdomen

**Table 2:** Biological properties of ADSCs.

Cell ID	Saturation Density (×10^6^/cm^2^)	Cloning Efficiency (%)
ADSC091809	1.39 ± 0.21	34.2 ± 3.1
ADSC020310	1.46 ± 0.15	33.9 ± 3.3
ADSC060210	1.47 ± 0.19	35.1 ± 2.7
ADSC121710	1.41 ± 0.14	34.6 ± 2.9
ADSC121708	1.41 ± 0.19	32.1 ± 2.7
ADSC100709	1.39 ± 0.17	33.1 ± 2.9
ADSC110409	1.41 ± 0.23	37.3 ± 4.1
ADSC012710	1.38 ± 0.18	35.6 ± 3.4

**Table 3:** Adipogenic differentiation of ADSCs.

Cell ID	BODIPY-Positive Cells (%)	Average FL/Cell
ADSC091809	96.9 ± 3.1	85.2 ± 6.7
ADSC020310	97.3 ± 2.7	80.1 ± 8.9
ADSC060210	98.4 ± 4.1	82.3 ± 7.7
ADSC121710	97.5 ± 3.9	84.6 ± 7.9
ADSC121708	97.3 ± 4.5	84.9 ± 5.1
ADSC100709	97.5 ± 3.7	81.0 ± 9.6
ADSC110409	97.4 ± 4.1	83.2 ± 7.5
ADSC012710	97.2 ± 3.9	86.7 ± 7.9

## Supplementary Materials

Supplementary materials can be found at https://www.mdpi.com/1422-0067/20/21/5505/s1.

## Abbreviations

ADSC	adipose-derived stem/progenitor cells
HIV	human immunodeficiency virus
HAART	highly active antiretroviral therapy
NRTI	nucleoside reverse transcriptase inhibitor
PI	protease inhibitor
UPR	unfolded protein response
d4T	2′,3′-didehydro-2′3′-dideoxythymidine
