# Supplementary material for: Biological Features Implies Potential Use of Autologous Adipose-Derived Stem/Progenitor Cells in Wound Repair and Regenerations for the Patients with Lipodystrophy
Source: Int J Mol Sci. 2019 Nov 5;20(21):5505. doi: 10.3390/ijms20215505 (PMC6862495; doi:10.3390/ijms20215505)
Supplement: Supplementary file 1 [file ijms-20-05505-s001.pdf]

Table S1 Summary of patients' therapies

Patient ID	Treatments
#1, #3	indinavir, lamivudine (3TC), d4T, atazanavir, Euzicom (3TC and abacavir combination)
#2	AZT, didanosine, 3TC, d4T, atazanavir, tenofovir/emtricitabine
#4	AZT, didanosine, lamivudine (3TC), nelfinavir, d4T, Euzicom (3TC and abacavir combination)
